# Clinical Diagnosis and Management Challenges of Harlequin Ichthyosis in a Preterm Neonate: A Case Report From Uganda

**DOI:** 10.1155/crdm/7982066

**Published:** 2025-01-21

**Authors:** Munanura Turyasiima, Djamila Magan Mohamed, Hamdi Mohamed Yusuf, Gloria Nakalema, Balbina Gillian Akot, Joan Kyoshabire, Shabirih Mutagamba, Grace Gladys Kimono, Jimmy Emmy Duca, Ibrahimu Makongwa

**Affiliations:** ^1^Department of Pediatrics and Child Health, Faculty of Clinical Medicine and Dentistry, Kampala International University, Kampala, Uganda; ^2^Department of Standards Compliance Accreditation and Patient Protection, Ministry of Health, Kampala, Uganda; ^3^Department of Management Science, Uganda Management Institute, Kampala, Uganda; ^4^Department of Dentistry and Oral Health, Amana Regional Referral Hospital, Ministry of Health, Dar es Salaam, Tanzania

**Keywords:** Africa, case report, diagnosis, harlequin ichthyosis, treatment

## Abstract

**Introduction:** Harlequin ichthyosis is a rare autosomal recessive genetic disorder resulting from mutations in the *ABCA12* gene. It is marked by distinctive skin abnormalities, including armor-like thickened scales separated by deep fissures. This condition is infrequently reported in the African population.

**Clinical Findings:** This report presents the case of a preterm neonate, born at 28 weeks of gestation, exhibiting dysmorphic features and severe generalized hyperkeratosis. The defining skin abnormalities included deep fissures across the head and trunk, bilateral eyelid ectropion, eclabium, underdeveloped auricles, and limbs enveloped in thick hyperkeratotic plaques with constricting bands and hypoplastic digits.

**Diagnosis, Interventions, and Outcomes:** The diagnosis of harlequin ichthyosis was established based on the characteristic clinical presentation. Supportive care included routine neonatal management and conservative treatment for prematurity-related respiratory distress syndrome. However, specific therapies, such as systemic retinoids, could not be administered due to their unavailability in the clinical setting. Unfortunately, the neonate passed away on the fifth day of life due to respiratory complications.

**Conclusion:** Harlequin ichthyosis remains associated with a high mortality rate, especially in resource-limited settings. Contributing factors include inadequate prenatal diagnostic services, restricted access to essential treatments, and insufficient neonatal care infrastructure, all of which exacerbate poor outcomes in developing countries.

## 1. Introduction

Low- and middle-income countries bear a disproportionate burden of congenital birth defects, leading to hundreds of thousands of associated deaths annually [1]. Harlequin ichthyosis (HI), a rare autosomal recessive genetic disorder caused by mutations in the *ABCA12* gene, represents the most severe form of congenital ichthyosis [2, 3]. Documented cases of HI are especially scarce within the African population.

The majority of neonates with HI do not survive beyond the first week of life due to complications such as respiratory failure, caused by restricted chest movements from the thickened scales, and sepsis, resulting from microbial infections through fissured skin [2–4]. In resource-limited settings, challenges such as inadequate laboratory diagnostic capacity and the unavailability of approved treatments for HI [5–7] hinder prenatal diagnosis and effective management.

This case report highlights the clinical features of HI in a newborn from Uganda, discusses available diagnostic approaches, and examines the management strategies employed in a Ugandan general hospital setting.

## 2. Case Presentation

### 2.1. Patient Information

A five-day-old female neonate was born at Kiryandongo General Hospital in Western Uganda at 28 weeks of gestation, with a birth weight of 1.6 kg. The baby cried immediately after birth and had reassuring APGAR scores of 7/10 at 1 min and 9/10 at 5 min.

The mother, a 21-year-old multiparous woman (G2P2), attended antenatal care only once. She was screened for HIV, syphilis, and hepatitis, all of which returned negative results. However, she did not take preconception folic acid during the first 12 weeks of pregnancy. No diagnostic ultrasounds were performed during the second or third trimesters, making it impossible to predict the presence of HI.

The parents denied consanguinity, and there was no known family history of ichthyosis or other genetic disorders. Additionally, the parents reported no significant radiation exposure during the pregnancy.

### 2.2. Clinical Findings and Diagnostic Assessment

#### 2.2.1. General Examination

We examined a dysmorphic, ill-appearing female preterm neonate with a Ballard score estimating 29 weeks of gestation. The neonate exhibited thick skin scales separated by deep fissures, generalized hyperkeratinization, ectropion, immature eyes and auricles, and flat fontanels. There was no cyanosis, pallor, or jaundice observed.

The neonate was in moderate distress, with bradycardia (heart rate of 98 bpm) and a respiratory rate of 73 breaths per minute. Although conscious, the neonate was hypothermic with a body temperature of 35.1°C. After being warmed using an infant warmer, the temperature normalized to 36.8°C.

Anthropometric measurements included a birth weight of 1.6 kg, a length of 45.3 cm, and a head circumference of 32.1 cm.

#### 2.2.2. Typical Clinical Features of HI on the Newborn

The diagnosis of HI in this neonate was based on characteristic clinical findings observed during a thorough head-to-toe examination of the newborn (Supporting Information Video 1).

##### 2.2.2.1. Skin

**Figure 1:**
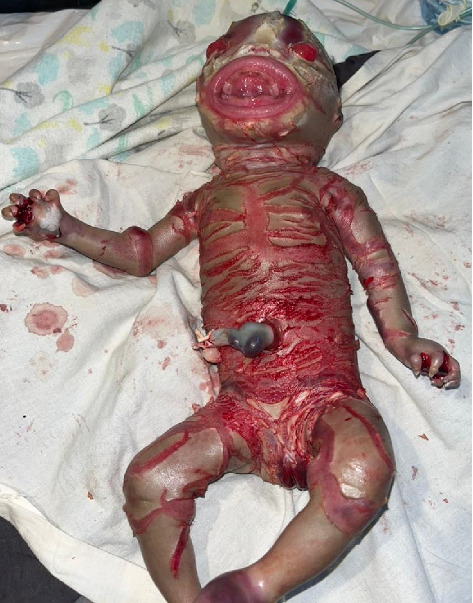
Severely thickened skin with shiny plates of hyperkeratotic scales.

**Figure 2:**
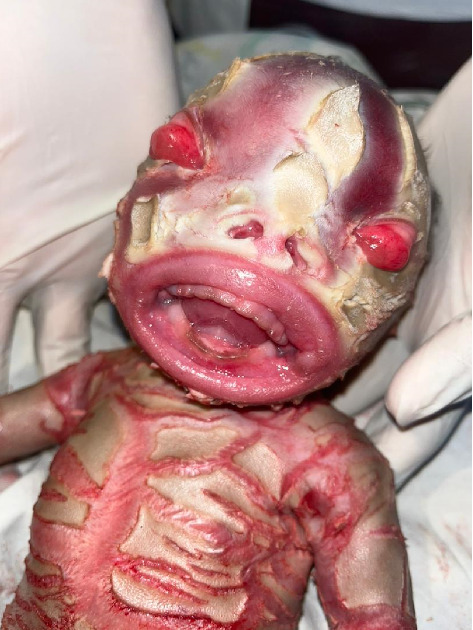
Severe ectropion and eclabium exposing the conjunctivae, cornea, and oral mucosa, respectively.

The neonate's skin appeared yellowish-gray, shiny, and thick, with hyperkeratotic scales separated by deep erythematous fissures covering the entire body. These features were more pronounced on the scalp, trunk, and abdominal areas, as demonstrated in Figures 1 and 2.

##### 2.2.2.2. Head

The head was small, with an occipitofrontal circumference of 32.0 cm. The scalp was covered with thick, black, shiny hair, and a diamond-shaped, erythematous hyperkeratotic fissure traversed through a membranous layer, as shown in Figure 2. Both the anterior and posterior fontanelles were flat.

##### 2.2.2.3. Face

**Figure 3:**
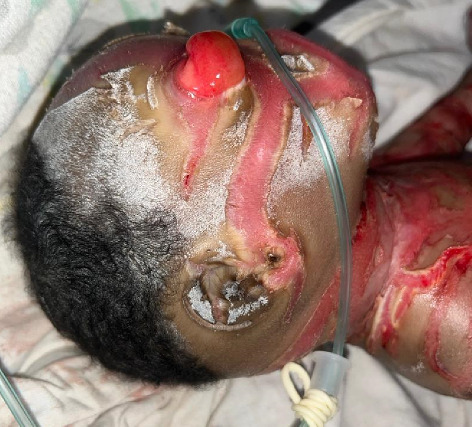
Severe ectropion and hypoplastic ears in harlequin ichthyosis.

The face was symmetrical. The eyes were open, with severe bilateral eyelid ectropion (eyelids turning outward from the eyeballs) and no discharge. Eyebrows and eyelashes were absent. The nose was hypoplastic with eroded alae and patent nares. The lips were everted, exposing the mucosal lining (eclabium), as illustrated in Figure 1. The retro-auricular folds and pinnae were rudimentary, small, and covered with a scaly membrane (Figure 3). The tongue was pink, smooth, and freely mobile without evidence of a tongue tie.

##### 2.2.2.4. Chest

The chest was symmetrical and moved with respiration, although the neonate exhibited respiratory distress (respiratory rate: 73 breaths per minute). The chest was covered with scaly plaques intersected by deep erythematous fissures. Bilateral air entry and breath sounds were normal upon auscultation.

##### 2.2.2.5. Abdomen

The abdomen appeared normally full and moved with respiration. The umbilicus was swollen and erythematous, but no discharge was observed.

##### 2.2.2.6. Limbs

**Figure 4:**
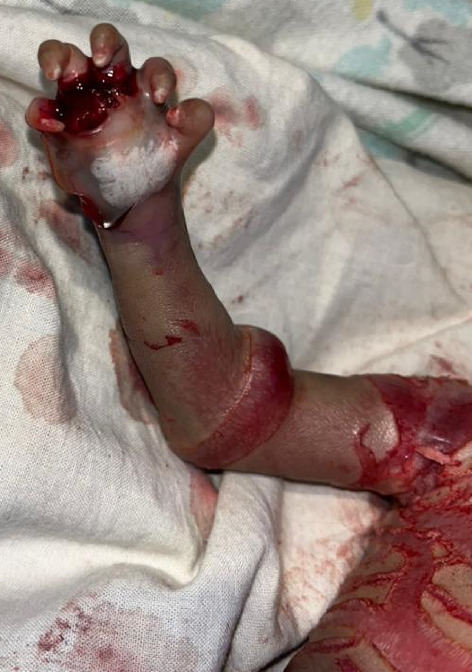
Right upper limb encased with thick hyperkeratotic skin with hypoplastic digits.

The upper and lower limbs were encased in thick, hyperkeratotic skin with constriction bands and hypoplastic digits. Compared to other body areas, the limbs had fewer fissures. The upper limbs were asymmetrical, with the right hand being smaller, contracted at the elbow, and flexed with contractures (Figures 1 and 4). The palms were swollen, erythematous, and lacked palmar creases. Both hands had five fingers with well-developed, claw-like nails. The lower limbs were symmetrical but fixed in flexion contractures. Ankles were markedly erythematous and swollen. Both feet had five toes with well-formed nails that were curved inward.

##### 2.2.2.7. Genitalia

**Figure 5:**
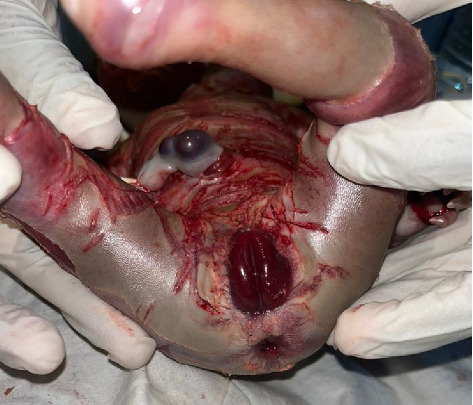
Genitalia in harlequin ichthyosis.

The external genitalia appeared female, with fused and edematous labia accompanied by prominent erythema. The clitoris was not visible, and no abnormal discharge or bleeding was noted (Figure 5). The anal opening was patent, and the neonate had passed meconium.

The findings of dysmorphism, prematurity, generalized hyperkeratotic skin with deep fissures on the head and trunk, severe bilateral eyelid ectropion, eclabium, hypoplastic auricles, and limbs encased in hyperkeratotic skin with constricting bands and hypoplastic digits are hallmark features of HI.

#### 2.2.3. Therapeutic Interventions

To stimulate fetal lung function and reduce the risk of cerebral palsy associated with prematurity, the mother was administered intrapartum dexamethasone (6 mg intravenous every 6 h) and a magnesium sulfate bolus (4 g intramuscular) for neuroprotection before cesarean delivery [8].

Management of the neonate primarily involved supportive care, as specific treatments for HI, such as moisturizers and topical or systemic retinoids, were unavailable in this setting.

Kiryandongo General Hospital operates a Level I Neonatal Intensive Care Unit (NICU) and lacks specialized resuscitation equipment, including continuous positive airway pressure (CPAP) machines, surfactant therapy for respiratory distress syndrome (RDS), neonatal apnea treatments such as caffeine citrate, and advanced feeding interventions like parenteral nutrition.

Immediately after birth, basic resuscitation measures were implemented, including gentle stimulation, drying, body warming on an infant warmer, oxygen therapy (1 L/min via an oxygen concentrator), and an intravenous bolus of 10% dextrose (5 mL/kg). These interventions successfully normalized the neonate's temperature, respiratory rate, and heart rate.

The neonate was subsequently initiated on routine newborn care, including fluid management with intravenous 10% dextrose and expressed breast milk (EBM), introduced on the third day of life. Prophylactic intravenous antibiotics, ampicillin (50 mg/kg every 12 h) and gentamicin (5 mg/kg once daily), were administered to prevent neonatal sepsis, a risk heightened by prematurity and skin barrier dysfunction in HI.

#### 2.2.4. Follow-Up and Outcomes

On the third day of life, supplemental feeding with EBM was initiated via a nasogastric tube (NGT) at 20 mL/kg/day, with a total fluid intake of 120 mL/kg/day. Despite receiving prophylactic treatment for apneas (intravenous aminophylline, 10 mg/kg loading dose over 20 min, followed by 5 mg/kg every 6 h), the neonate continued to experience episodes of uncontrolled apneas.

On the fourth day, the neonate's respiratory distress worsened with severe tachypnea (average respiratory rate of 78 breaths per minute), and apneas became more frequent. The medical team withheld NGT feeding and continued intravenous fluids and oxygen therapy via nasal prongs. Unfortunately, the baby passed away on the fifth day of life due to respiratory complications associated with prematurity.

Throughout the course of care, socioeconomic challenges and the social stigma surrounding the birth of a child with dysmorphism [6] led to instances where the parents were reluctant to accept medical treatment, despite multiple counseling attempts. The mother also faced difficulties in consistently providing EBM to the nursing team at the required times.

## 3. Discussion

This case presents an African newborn with typical features of HI, a rare autosomal recessive genetic disorder caused by mutations in the *ABCA12* gene [3, 9]. While HI affects individuals across all ethnic groups, it is associated with high morbidity and mortality during the first week of life, even with advancements in treatment options.

### 3.1. Diagnosis and Clinical Presentation

The diagnosis of HI is typically made through postnatal examination, where the newborn's characteristic skin features are evident. However, it can also be diagnosed through antenatal ultrasound, DNA testing, and skin biopsy histopathological analysis [5, 9–11]. Given its autosomal recessive inheritance pattern, a positive family history of genetic disorders commonly seen in ichthyoses should prompt genetic counseling and testing, along with further diagnostic evaluations.

Antenatal scanning, particularly in the second and third trimesters, preferably using three-dimensional and four-dimensional sonography, can reveal abnormal facial features such as ectropion (eversion of the eyelids), eclabium (eversion of the lips), shortened foot length, incurved toes, clenched fists, poor delineation of nostrils, and polyhydramnios. These findings are pathognomonic of HI [5, 10]. Prenatal diagnosis through DNA testing can identify mutations in the ABCA12 gene, and chorionic villus sampling (CVS) or amniotic fluid cell analysis can confirm the condition, especially in mothers with a history of previous children diagnosed with ichthyoses [5, 10]. However, such tests are often unavailable or unaffordable in many developing countries [6, 12].

In this case, prenatal diagnosis was not possible, as the mother did not access antenatal ultrasound, and DNA and histology testing were not available at the hospital. Additionally, the family faced significant financial challenges.

Similar to many other congenital ichthyoses, neonates with HI are born with thick, armor-like skin plates covering their entire body [4]. The clinical presentation is characterized by truncal plates with fissuring, bilateral ectropion (eversion of the eyelids), eclabium (eversion of the lips), and constricting bands with contractures on both the upper and lower limbs [3, 9, 11]. Such infants are typically preterm and often succumb within the first week due to complications such as respiratory distress and infection [2, 3]. This neonate, born prematurely at 28 weeks of gestation, displayed all the hallmark features of HI (Figures 1, 2, 3, 4, and 5).

In addition to the skin manifestations, HI infants develop respiratory distress and later respiratory failure due to the constricting effect of the thickened keratinized skin, which impairs proper chest expansion and movement of the respiratory muscles [2]. Decreased lung expansion increases the risk of amniotic fluid aspiration and neonatal pneumonia. The exposure of skin to the environment leads to excessive water loss, increasing the risk of dehydration [2, 4, 11]. Skin erosions further predispose the infant to bacterial and fungal infections, which may result in sepsis. Furthermore, compartment syndrome can develop due to the tightness of the hyperkeratotic skin, constricting the digits and distal limbs, leading to ischemia [2, 3, 9, 11].

### 3.2. Available Treatment Options

There is currently no approved cure for ichthyoses, and management remains primarily supportive, focusing on rehydration, eye care, and skin care tailored to the patient's age [2]. A multidisciplinary approach is essential, involving neonatologists, ophthalmologists, dermatologists, and nutrition specialists [2], with an emphasis on skin protection and infection prevention [4].

Neonatal management of HI includes placing the infant in a humidified incubator with close monitoring of homeostasis in the NICU. Continuous clinical surveillance for bacterial and fungal infections, pneumonia, sepsis, and compartment syndrome is crucial. Frequent application of moisturizing skin ointments is also recommended. In severe cases, systemic acitretin may be considered [2].

#### 3.2.1. Feeding and Nutrition

Neonates with HI often face significant feeding challenges due to thick scaling around the jaw. Early consultation with a dietitian is essential, and placement of a nasogastric feeding tube is recommended to meet the increased caloric demands. As the infant grows, a gastrostomy tube may be necessary to ensure adequate nutrition [2, 11]. If sun exposure is limited, vitamin D supplementation is also advised.

#### 3.2.2. Eye and Skin Care

Eye care for neonates with HI involves topical periocular retinoid treatment. Given the fragile and exposed nature of the skin, it is crucial to protect it from temperature extremes. Regular application of moisturizers such as humectants (glycerin and propylene glycol), retinoids, and emollients (petrolatum) can help promote epidermal differentiation and barrier integrity in cases of moderate to severe hyperkeratosis [2].

Topical retinoids (e.g., tazarotene cream) and oral retinoids (e.g., acitretin, dosage: 0.25–1 mg/kg/day) are available treatment options [2, 3, 11]. However, these should be administered in low doses with appropriate anticipatory guidance to minimize side effects. Due to their high cost and limited availability in resource-limited settings, these treatments were not feasible in this case.

#### 3.2.3. Bathing and Scale Removal

Daily bathing helps hydrate and cleanse the skin, with products like alkaline sea water or baking soda recommended for 30–60 min weekly to aid in scale removal. Additionally, dilute bleach baths (using acetic acid or vinegar) can help reduce the burden of bacterial and fungal skin infections [2].

Scale removal can be further enhanced through long baths followed by gentle physical exfoliation with a soft washcloth or fine-toothed comb. Chemical keratolytics, such as emollients containing urea, lactic acid, salicylic acid, and glycolic acid, have also been successfully used, but they require caution when used in infants and young children [2]. In some cases, skin transplants may offer a promising treatment option, though the long-term outcomes are not yet fully understood [2].

#### 3.2.4. Psychosocial and Genetic Counseling

Parents of children with congenital abnormalities, particularly in developing countries, face significant socioeconomic challenges and social stigma [6, 7]. Addressing these issues through psychosocial support and genetic counseling is essential. In this case, the parents initially requested that treatment be withheld, but after counseling (without genetic testing), they agreed to allow medical intervention. Unfortunately, the neonate succumbed to respiratory complications of prematurity on day five of life.

### 3.3. Outcomes and Prognosis

The majority of HI cases result in death within the first week of life, primarily due to complications associated with prematurity, which is strongly linked to this condition [4, 10]. In this particular case, the neonate succumbed to prematurity-associated respiratory distress on the fifth day of life.

Over the past decades, survival rates for HI have improved significantly, thanks to increased awareness of the condition, advances in neonatal care, and the potential benefits of early treatment with oral retinoids [2, 7]. Infants who survive beyond the neonatal period often exhibit skin changes resembling severe nonbullous congenital ichthyosiform erythroderma [3] and face numerous long-term complications. These include persistent ectropion, juvenile idiopathic arthritis, motor developmental delays [11], and other challenges.

Continuous monitoring of growth and development is essential for survivors, along with comprehensive care strategies such as ensuring adequate nutrition, including vitamin D supplementation, and implementing rigorous topical skincare routines.

## 4. Conclusion

In resource-limited settings, postnatal diagnosis of HI remains crucial and is primarily based on the characteristic armor-like appearance of the skin at birth. Prenatal diagnosis, which involves antenatal imaging and confirmation through DNA genetic testing, is often not feasible in these settings due to the lack of testing laboratories and financial constraints.

### 4.1. Methods

This case report was prepared following the CARE Clinical Case Report Guidelines [13].

## Data Availability

The data that support the findings of this study are available on request from the corresponding author. The data are not publicly available due to privacy or ethical restrictions.

## Supporting Information

The video of a preterm neonate showing typical features of harlequin ichthyosis (Video. 1).
